# SOCIAL RELATIONSHIPS AND OBESITY IN LATER LIFE: A LONGITUDINAL ANALYSIS

**DOI:** 10.1093/geroni/igz038.734

**Published:** 2019-11-08

**Authors:** Jane L Tavares, Jeffrey Burr, Edward A Miller

**Affiliations:** 1 University of Massachusetts Boston Gerontology Institute, LeadingAge LTSS Center, Boston, Massachusetts, United States; 2 University of Massachusetts Boston, Boston, Massachusetts, United States

## Abstract

The majority of U.S. older adults are overweight or obese. Social relationships are a key factor linked to obesity among younger age groups, but there are no known investigations of these modifiable risk factors older for adults. This study examined the association between quantitative and qualitative indicators of social relationships and waist circumference among middle-aged and older adults. We also examined whether psychosocial and health behavior characteristics mediate and/or moderate these relationships. Using the 2006 and 2010 waves of the Health and Retirement Study, a series of regression models were estimated to examine the longitudinal association between social relationships and waist circumference in age stratified samples (age 50 to 64; age 65 and older). Results for those age 50 to 64 indicated that higher positive social support and lower negative social support were associated with lower waist circumference over time. For those age 65 and older, lower negative social support and higher loneliness were associated with lower waist circumference over time. Further, daily exercise and anxiety were observed to be mediators of these associations; both variables also acted as moderators. Findings from this study highlight the need for healthcare providers to assess older adults’ levels of social support and loneliness in order to achieve a more comprehensive understanding of wellness. The results also underscore the importance of developing intervention programs that provide widespread and ample opportunities for older adults to engage socially, particularly those that incorporate or encourage physical activity and healthy eating.

